# Onset of Adverse Abdominal Events Due to Intestinal Ischemia-Reperfusion Injury after Aortic Cross-Clamping Is Associated with Elevated HSP70 Serum Levels in the Early Postoperative Phase

**DOI:** 10.3390/ijms232315063

**Published:** 2022-12-01

**Authors:** Panagiotis Doukas, Gabriel Hellfritsch, Hanif Krabbe, Jelle Frankort, Michael J. Jacobs, Alexander Gombert, Florian Simon

**Affiliations:** 1 European Vascular Center Aachen-Maastricht, Department of Vascular Surgery, RWTH University Hospital Aachen, 52074 Aachen, Germany; 2 Clinic for Vascular and Endovascular Surgery, University Hospital Duesseldorf, Heinrich-Heine-University Duesseldorf, 40225 Düsseldorf, Germany

**Keywords:** ischemia reperfusion, intestinal ischemia, paralytic ileus, aortic surgery, postoperative management, cell dysfunction

## Abstract

Tissue injury of the viscera during open thoracoabdominal aortic (TAA) reconstructions has been reported as the aftermath of the ischemia-reperfusion mechanism following supracoeliac aortic cross-clamping. Abdominal complications after open aortic reconstructions, although rare through the intraoperative implementation of selective visceral artery blood perfusion, are associated with high rates of reinterventions and a poor prognosis. Recent animal experiments demonstrated that provoking mesenteric ischemia in rats induces the leukocyte-mediated transcription of heat-shock protein 70 (HSP70), a chaperone belonging to the danger-associated molecular pattern proteins (DAMPs). Translating these findings clinically, we investigated the serum levels of HSP70 in patients undergoing open aortic reconstructions with supracoeliac clamping. We postoperatively observed a relevant induction of HSP70, which remained significantly elevated in cases of postoperative abdominal complications (paralytic ileus, abdominal compartment syndrome, and visceral malperfusion). The receiver–operator curve analysis revealed the reliable prognostic accuracy of HSP70 as a biomarker for these complications as soon as 12 h post-operation (AUC 0.908, sensitivity 88.9%, specificity 83.3%). In conclusion, measuring HSP70 serum levels in the early postoperative phase may serve as a further adjutant in the diagnostic decision making for both the vascular surgeon and intensivist for the timely detection and management of abdominal complications following open TAA surgery.

## 1. Introduction

Supracoeliac aortic cross-clamping during open thoracoabdominal aortic reconstructions (TAA) interrupts the natural, pulsatile flow to the visceral organs, resulting in reduced oxygen supply to the cells, which in return fail to meet their own energy requirements [1]. The restoration of the blood-flow may aggravate the effect of ischemic injuries through the formation of oxygen species (ROS) [2]. Damage to the intestinal mucosa through reperfusion may persist for days after the ischemic event. The cellular reaction to the oxidative stress induced through ischemia and reperfusion translates to systemic dysfunction and apoptosis on the cellular level and in local and remote tissue inflammation [1].

The manifestation of an ischemia-reperfusion injury (IRI) of the intestinal wall cells can vary in its clinical severity. Abdominal complications associated with dysfunction and apoptosis of the intestinal cells may span from a temporary motility reduction of the gut to ischemia of entire intestinal segments. Complications like abdominal compartment syndrome or paralytic ileus may be less limiting on patients’ prognosis but may still prolong the hospital stay and complicate the postoperative phase. Intestinal ischemia on the other hand, although rare with an incidence of less than 10%, is associated with a relevant mortality and morbidity rate [3]. Thus, the timely detection of abdominal complications remains a relevant issue in the postoperative management of patients after open TAA reconstructions, and it currently largely relies on a clinical assessment, radiographic signs, and bloodwork parameters—lactate being the most commonly used one. The diagnostic capacity of lactate, however, is limited through its low specificity and late response to mesenteric damage [4].

Heat-shock protein 70 (HSP70) is part of the chaperone family and plays an essential role in regulating intracellular protein homeostasis [5]. The HSP family is counted under the danger-associated molecular pattern molecules (DAMPs), the expression of which is enhanced in injured or otherwise stressed cells [6] as a measure of self-protection through the modulation of inflammation [7]. Animal experiments have described an elevation of HSP70 in an effort to protect mucosal integrity after intestinal IRIs in a rat model [8], and a recent study on the expression of HSP70 during visceral IRIs in rats found a significant correlation between HSP70-mRNA induction and the severity of the IRI [7].

In order to support the diagnostic decision algorithm in the early postoperative phase, we investigated the accuracy and reliability of persisting high heat-shock protein 70 (HSP70) levels in a serum as a potential biomarker for abdominal complications to predict adverse abdominal events after an intestinal ischemia-reperfusion injury.

## 2. Results

The mean age of the 21 included patients (6 women) was 50.6 ± 12.4 years. The patient demographic data and procedural details are demonstrated in Table 1. There was no significant correlation between these characteristics, including aortic cross-clamping time and the onset of abdominal correlations.

**Figure 1:**
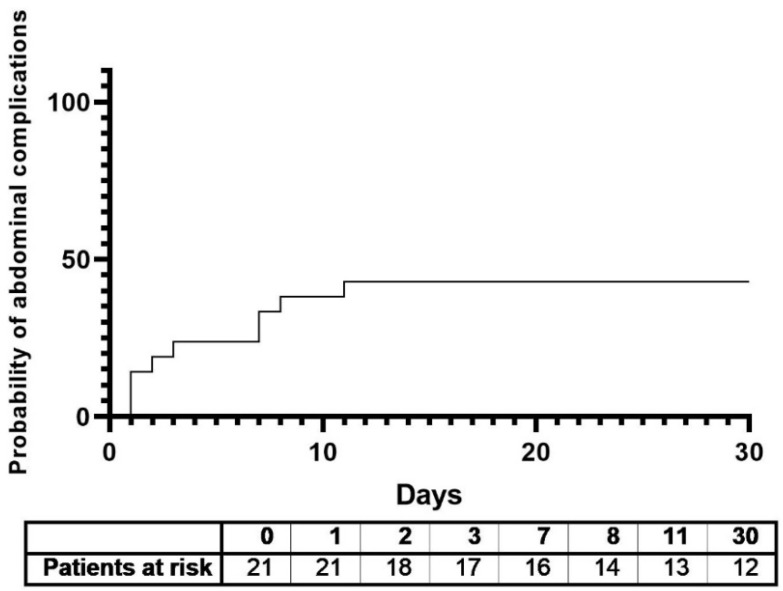
Kaplan–Meier plot. Probability of abdominal complications in the first 30 days after surgery.

Nine patients (42.9%) developed at least one of the following abdominal complications: paralytic ileus (9.5%), abdominal compartment syndrome (9.5%), or visceral malperfusion (23.8%). The mean time after surgery for the onset of abdominal complications was 4.8 ± 1.3 days (Table 2) (Figure 1). The rate of operative revisions and reinterventions was significantly higher in the abdominal complications group (r = 0.44, *p* = 0.046), which also significantly correlated with the rate of revision laparotomies (r = 0.62, *p* = 0.003), and in two cases, intestinal resection was necessary. The patients in the abdominal complications group also required significantly more days of catecholamine support in order to sustain normal blood pressure levels (17.9 ± 12 vs. 6 ± 6.4, r = 0.56, *p* = 0.013). There was no significant difference in the 30-day mortality and rates of sepsis between the two groups, although deaths and septic events occurred more frequently in the abdominal complications group. Patients with abdominal complications had a significantly longer period to discharge from the hospital (hospital stay days: 30.9 ± 15.6 vs. 62.7 ± 41.1, r = 0.49, *p* = 0.02) and a tendency for a prolonged stay in the intensive care unit. The postoperative characteristics and complications are presented in Table 2.

### 2.1. Persisting Elevation of HSP70 Serum Levels in Patients with Abdominal Complications

**Figure 2:**
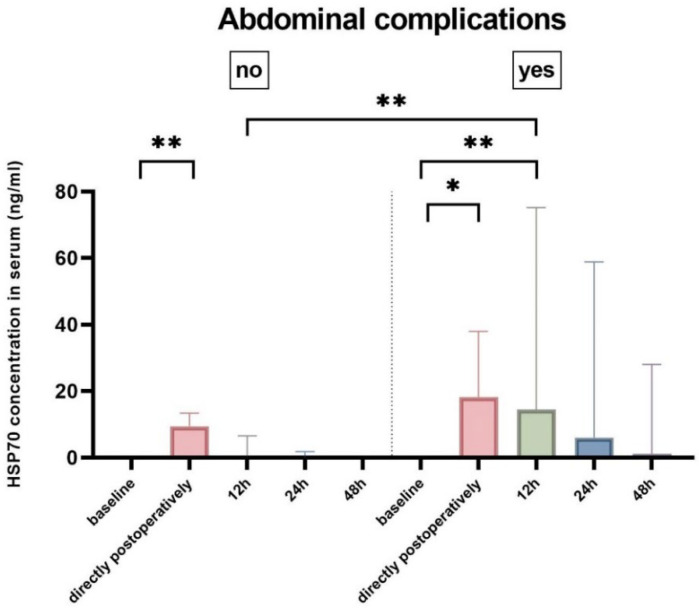
Heat-shock protein 70 (HSP70) serum levels at different time points for the two groups: abdominal complications (*n* = 9) and no abdominal complications (*n* = 12). Bar represents the median, and the whiskers represent the interquartile range. Significance is annotated above the brackets as (*) for *p* < 0.05 and (**) for *p* < 0.01.

In all patients, HSP70 serum levels were significantly elevated directly after surgery (median difference to baseline: 11.77 ng/mL, *p* < 0.001) and remained significantly elevated until 12 h after surgery (median difference to baseline: 7.59 ng/mL, *p* = 0.002), however with a tendency to recovery (Table 3). We observed an elevation of HSP70 serum levels at all postoperative time points in patients who later developed abdominal complications with a statistically significant increase at 12 h after surgery (14.5 ng/mL vs. 0 ng/mL, *p* = 0.007) (Table 3) (Figure 2). HSP70 levels did not significantly correlate with the aortic cross-clamping time (Supplementary Table S1). The course of HSP70 serum levels for each case is plotted in Supplementary Figure S1.

In the subgroup of patients with visceral malperfusion (n = 5), a univariable logistic regression analysis revealed a significant correlation of HSP70 serum levels at 12 h (rho = 0.51, *p* = 0.05) after surgery (Table 4) (Supplementary Figure S2). Abdominal compartment syndrome and paralytic ileus did not significantly correlate with HSP70 serum levels.

### 2.2. HSP70 Levels as a Biomarker for Abdominal Complications

**Figure 3:**
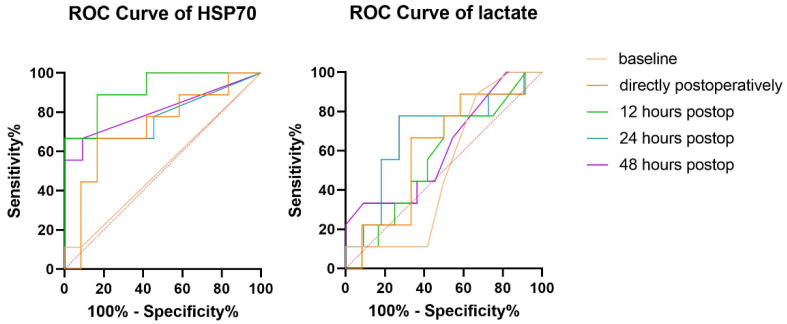
ROC curves for heat-shock protein 70 (HSP70) serum levels and lactate.

ROC analysis revealed reliable diagnostic accuracy for HSP70 levels as a biomarker for abdominal complications at all postoperative time points (Table 5) (Figure 3). The largest area under the curve (AUC) was achieved at 12 h after surgery (AUC 0.909, *p* = 0.002, sensitivity 88.9%, specificity 83.3%) for a cut-off value of 7.6 ng/mL. On the other hand, lactate levels (mmol/L) reached the highest AUC at 24 h after surgery (AUC 0.687, *p* = 0.141, sensitivity 77.8%, specificity 78.7%), which was still lower than that of the HSP70 serum levels. The overall positive predictive value (PPV) of HSP70 serum levels was 80% and the negative predictive value (NPV) was 90%.

## 3. Discussion

Cells in hypoxic conditions switch their mitochondrial metabolism from aerobic to anaerobic in order to attain the necessary adenosine triphosphate (ATP) production. Anaerobic metabolism is less efficient in the ATP production and formation of antioxidative agents than its aerobic counterpart. Furthermore, lactic acid is formed as a by-product of anaerobic metabolic activity and accumulates in the cytoplasm, decreasing cellular pH. Acidosis compromises enzyme activity and the effectiveness of electrolyte membrane pumps, leading to a hyperosmolar cell state followed by osmosis-driven cell swelling [1].

The reinstatement of oxygen supply to the cell increases the generation of ROS, which cannot be adequately eliminated through the remaining antioxidant resources of the cell. The resulting oxidative stress promotes tissue inflammation and dysfunction, which may eventually lead to cell death [9]. The severity of cell damage due to reperfusion can greatly vary and it is directly dependent upon the duration of the ischemia [10]; although a prolonged IRI may end in cell necrosis, moderate IRIs may only temporarily inhibit the physiologic functions of the cell, as the focus of its synthetic activity turns to survival measures [11]. A reperfusion injury is a dynamic process which may persist for days after the ischemic event.

Despite intraoperative protective strategies to reduce splanchnic hypoperfusion during open TAA surgery with supracoeliac cross-clamping—such as distal aortic perfusion (DAP) and selective visceral perfusion (SVP), as well as reducing metabolic activity of the gut wall through mild systemic hypothermia [12]—intestinal wall cells experience IRIs. The subsequent inflammatory response may further aggravate the effects of hypoperfusion [13] and the manipulation of the splanchnic neural network, compromising gut motility and prolonging in this way the clinical presentation of paralytic ileus. Intestinal anergia is typically diagnosed through clinical assessment and/or radiographic imaging and is conservatively managed in most cases [14]. However, progressive bowel distension with increased intrabdominal pressure may further complicate the postoperative phase and require surgical revision.

A rare but potentially lethal gastrointestinal complication after open TAA surgery is irreversible gut ischemia (incidence 5%, mortality 55–60%) [15]. Hypoperfusion of the splanchnic circulation as well as occlusive thromboembolic events lead in many cases to the resection of the necrotic intestinal segments. Due to bacterial translocation through the gut wall and the accompanying systemic inflammatory response, patients require prolonged catecholamine support for maintaining normal blood pressure. Our results confirm this clinical experience; abdominal complications significantly correlated with the re-intervention rate (*p* = 0.043) and days with catecholamine support (*p* = 0.013). The reintervention rate presented in this study (52.4%) summed all minor and major revisions in this cohort. Under the most frequent reintervention “re-thoracotomy”, we summarized all thoracic revisions, including the thoracoscopic evacuations of hematomas. Under “wound complications”, we included wound-conditioning procedures with vacuum-assisted closure therapy. In a recent publication of our group, the aortic related reintervention rate after open TAA reconstructions was reported at 2.8% [16]. Nevertheless, the abdominal complications group was significantly associated with the relaparotomy rate (0% vs. 44.4%, *p* = 0.003) and we observed a trend for 30-day mortality (8.3% vs. 33.3%, *p* = 0.164) and also for sepsis (16.7% vs. 55.6%, *p* = 0.066). The incidence of visceral malperfusion reported in our study (23.8%) coincided in only two cases with irreversible gut ischemia (9.5%).

Studies in mice and humans have demonstrated that the inflammatory response after supracoeliac aortic cross-clamping and the subsequent IRI have systemic consequences [7,17,18]. In the rat models, 30 min of supracoeliac aortic cross-clamping led to a significant increase in serum levels of diverse factors (tumor necrosis factor alpha (TNFa)) and interleukin ((IL)-1β und IL-10) and was proven lethal for the animal [17]. In open TAA repair patients, IL-10 levels in the serum were dependent upon the aortic cross-clamping time and correlated with organ dysfunction [18]. Enhanced levels of circulating intestinal fatty acid-binding proteins (iFABPs) at the end of open aortic surgery have been shown to accurately predict the onset of postoperative intestinal ischemia [19]. Yet, the need for a biomarker for intestinal injuries is still present [20].

Mine et al. recently investigated the effect of intestinal IRIs on the transcription of HSP70 in rat leukocytes and found that an extensive IRI led to significantly elevated HSP70 transcription [7]. In line with these findings are our observations in patients with abdominal complications after open TAA repair; 12 h after surgery, HSP70 serum levels were significantly higher in patients with abdominal complications (14.5 vs. 0, *p* = 0.007) and remained elevated in the subsequent measures, although without statistical significance. The subgroup analysis for visceral malperfusion events revealed a correlation with HSP70 serum levels at all postoperative time points with a statistical significance at the 12 h (*p* = 0.02) and 48 h marks (*p* = 0.03). We speculate that the elevation at the 24 h mark was not found to be significant due to the small sample size. Nevertheless, we could describe a trend for this measure point (*p* = 0.07).

Directly postoperatively, we observed a significant elevation of HSP70 serum levels compared with baseline levels for all patients (baseline 0 vs. postop 11.77, *p* < 0.001) with a trend for higher values in the abdominal complications cohort (18.3 vs. 9.5, *p* = 0.10). A possible explanation for the directly postoperative elevation of HSP70 serum levels is the stress induced through the operative trauma. Extensive trauma has been suggested in the literature as a trigger for systemic inflammation and a reactive induction of HSPs [21]. HSP serum levels are further increased if the trauma involves the thoracic region. Given the considerable tissue injury from the thoracoabdominal exposure for the aorta, one could hypothesize a similar mechanism of early HSP induction.

In order to evaluate the diagnostic accuracy and reliability of HSP70 serum levels as a biomarker for abdominal complications, we performed an ROC analysis. Twelve hours after surgery, the test reached its maximum diagnostic capacity (AUC = 0.909, *p* = 0.002, sensitivity 88.9%, specificity 83.3%). The test demonstrated high specificity at all postoperative time points with an overall negative predictive value of 90%. In comparison, conventional lactate measurements in patients’ serums at the same time points showed an inferior predictive ability for abdominal complications with its peak AUC at 24 h after surgery (AUC = 0.687, *p* = 0.141, sensitivity 77.8%, specificity 78.7%). Summarizing these findings, we observed an advantage in the diagnostic ability of HSP70 compared with the long-established lactate in terms of both early detection (12 h vs. 24 h) and reliability (sensitivity 88.9% vs. 77.8%). The combination of HSP70 with other biomarkers for intestinal ischemia, such as iFABP, could potentially reinforce the prognostic accuracy of HSP70 even further and remains an interesting field for future investigation.

### Strengths and Limitations

This study was limited by the small number of included patients and strong skewedness of the data for HSP70 measurements. In order to compensate for this, a nonparametric statistical analysis was performed and the conservative variants of post hoc tests were chosen. Moreover, the substantial effect size reinforces the significance of the observed differences. Due to the small cohort size, we could also not elucidate any significant differences between genders. On the other hand, two strong points of this research lied in patient inclusion and in the surgical procedure; all patients were operated on by the same surgical team with a standardized protocol, reducing possible performance bias. Another limitation of this project lied in the heterogeneity of the investigated endpoints; although the underlying pathomechanism of the assessed abdominal complications was similar, its clinical impact has a wide range. Yet, this study was to our knowledge the first to investigate HSP70 in the field of thoracoabdominal aortic surgery and may function as a pilot for larger trials, possibly also in clinical disciplines other than vascular surgery. The external validation of the hypothesis of this study in other centers and in prospective, multicenter studies may be a first step in evaluating the transferability of our findings.

## 4. Materials and Methods

### 4.1. Study Design

Twenty-one patients undergoing elective, open TAA reconstructions from January 2019 to December 2021 were recruited in this prospectively conducted, single-center observational study, which was reviewed by the ethics committee of the University Hospital Aachen (EK004/14). Inclusion criteria were aortic aneurysms involving both the thoracic and the abdominal aortas, requiring reconstruction of the viscero-renal segment. The project was designed according to the Declaration of Helsinki and the STROBE criteria. Written informed consent was obtained from all patients before inclusion. Pregnant women and patients younger than 18 years of age were excluded. Data on patients’ medical history, demographic characteristics, as well as clinical charts were collected from digital medical records.

### 4.2. Surgery

The detailed surgical protocol for TAA reconstructions has been previously described in the literature [22]. All cases required the reconstruction of the viscero-renal aortic segment. Distal aortic perfusion was achieved via extracorporeal circulation through femoral cannulation, and the selective perfusion of all visceral arteries was initiated after the target vessels were directly cannulated. Perfusion catheters with inflatable tips and pressure channels were used for the cannulation of the superior mesenteric artery and celiac trunk, providing pressure-controlled blood flow. Both kidney arteries were infused with a maximum of 1L CUSTODIOL^®^ solution (HTK, Dr. Franz Köhler Chemie GmbH, Bensheim, Germany) per kidney, and the spinal cord integrity was evaluated through the intraoperative monitoring of motor-evoked potentials [23]. A decrease in the amplitude of the motor-evoked potentials indicated compromised spinal cord function and triggered the cannulation and selective perfusion of back-bleeding intercostal arteries, which were later included in the aortic reconstruction.

### 4.3. HSP70 Measurements

Serum samples from patients’ blood were directly collected postoperatively at baseline 12, 24, and 72 h after surgery. The samples were centrifuged at 4 °C (1500 rcf for 10 min) and the supernatants were retrieved and stored at −80 °C. HSP70 concentrations in the serum were calculated through an enzyme-linked immunosorbent assay (ELISA) using a commercial human HSP70 ELISA kit (Catalog Nr. SEA873Hu, Cloud Clone Corp, Houston, TX, USA). The intra-assay coefficient of variation was 12%, and the inter-assay coefficient was 8%. Assays exceeding these values were repeated.

### 4.4. Endpoints

The primary objective of this study was to evaluate the diagnostic accuracy of HSP70 as a serum biomarker for abdominal complications after TAA surgery. Under the term abdominal complications, we investigated the incidences of paralytic ileus, abdominal compartment syndrome, and visceral malperfusion. Paralytic ileus was diagnosed when at least two of the following criteria were met after the fourth postoperative day: absence of flatus over the previous 24 h, abdominal distension, and radiologic confirmation [24]. Abdominal compartment syndrome was defined when the intra-vesicular pressure exceeded 20 mmHg [25]. Visceral malperfusion was diagnosed in patients with an acutely onset, partial or complete, thrombotic occlusion of the superior mesenteric artery and/or coeliac trunk, as revealed in an angiographic scan. Not all patients with visceral malperfusion required surgical revision. Patients with at least one of these abdominal complications were included in the “abdominal complications” group. If a patient had more than one abdominal complication, he/she was counted once for the most severe one (visceral malperfusion being more severe than abdominal compartment syndrome being more severe than paralytic ileus).

Secondarily, preoperative patient characteristics and procedural details (Table 1) were investigated for risk factors for the onset of postoperative abdominal complications. Potential correlations of abdominal complications and other postoperative effects were also investigated as follows: if the daily sequential organ failure assessment (SOFA) score of a patient was increased by 2 points or more compared with the previous day and an active infection was suspected, then the patient was considered septic [26]. Patients with an acute onset of jaundice and a spontaneous international normalized ratio (INR) > 1.5 were diagnosed with acute liver failure [27]. Kidney injury was assessed according to the KDIGO criteria [28], and a newly onset need for dialysis (KDIGO 3) was considered a relevant postoperative complication. The diagnosis for acute respiratory distress syndrome (ARDS) was met according to the Berlin definition [29], and severe hypoxemia (ARDS 3) was considered a relevant postoperative complication. Lastly, the failure of two or more vital organ systems fulfilled the diagnosis criteria for multi-organ dysfunction syndrome (MODS) [30].

### 4.5. Statistics

Categorical variables were reported as absolute frequencies and percentages of the whole sample. Continuous variables are presented as mean (±standard deviation) or in the case of extensive skewedness of the data, as median (interquartile range). Significance levels are marked with (*) for *p* < 0.05, (**) for *p* < 0.01, and (***) for *p* < 0.001 with a 95% confidence interval (CI). Patients’ characteristics were postoperatively tested for an association with the onset of abdominal complications using the odds ratio (OR) in the case of categorical variables and with a univariable logistic regression analysis with Firth’s bias correction in the case of continuous variables. A further univariable logistic regression analysis with Firth’s bias correction was performed for the correlation testing of postoperative complications with abdominal complications. An elevation of HSP70 levels for all patients was tested for significance using the Friedman’s two-way analysis of variance by ranking related samples with the Bonferroni correction. A correlation between HSP70 serum levels and abdominal complications was tested for significance using the Kruskal–Wallis test for nonparametric data, followed by the post hoc Dunn’s test for multiple comparisons. The probability of abdominal complications within the first 20 days of surgery was estimated with a survival analysis, graphically presented with a Kaplan–Meier curve. The accuracy of HSP70 serum levels and lactate as biomarkers for abdominal complications was assessed using a receiver-operating characteristic analysis (ROC). The optimal cut-off value was calculated using the Kolmogorov–Smirnov test. The data was analyzed using SPSS software v27 (SPSS Inc., Chicago, IL, USA) and graphically demonstrated using GraphPad Prism version 8.0.0 for Windows (GraphPad Software, San Diego, CA, USA).

## 5. Conclusions

In conclusion, an elevation of HSP70 serum levels could reliably predict the onset of postoperative abdominal complications in our cohort as soon as 12 h after surgery—earlier and more accurately than lactate serum levels, which are commonly used in standard clinical practice. We speculate that very high levels of HSP70 after the first 12 h after surgery might justify early imaging of the abdomen and visceral vessels. Thus, monitoring HSP70 serum levels in the early postoperative phase may serve as a further adjutant in the diagnostic decision making for both the vascular surgeon and intensivist for the timely detection and management of abdominal complications following open TAA surgery.

## Figures and Tables

**Table 1:** Demographic data and procedural details. Values are the number (%) or mean ± SD, as indicated. Correlations are reported with the odds ratio and a 95% confidence interval for categorical variables; however, those indicated with ^†^ are continuous variables, which are reported with Pearson’s (r) and *p*-value; BMI: body mass index; COPD: chronic obstructive pulmonary disease; CKD: chronic kidney disease.

	All (*n* = 21)	No Abdominal Complications (*n* = 12)	Abdominal Complications (*n* = 9)	Correlations
Age (years)	50.6 ± 12.4	51.7 ± 11.3	49.1 ± 14.3	−0.1 (*p* = 0.65) ^†^
Women	6 (28.6)	3 (25)	3 (33.3)	0.67 [0.1–4.48]
Smoking	7 (33.3)	4 (33.3)	3 (33.3)	1 [0.16–6.25]
Obesity	3 (14.3)	1 (8.3)	2 (22.2)	3.14 [0.24–41.5]
BMI (m/kg^2^)	24.2 ± 4.2	24.1 ± 3.7	24.4 ± 5	0.45 (*p* = 0.85) ^†^
COPD	4 (19)	2 (16.7)	2 (22.2)	1.43 [0.16–12.7]
Hypertension	18 (81)	8 (66.7)	9 (100)	0.67 [0.45–0.1]
CKD	12 (57.1)	7 (58.3)	5 (55.6)	0.89 [0.16–5.11]
Prior aortic procedures	13 (61.9)	8 (66.7)	5 (55.6)	0.63 [0.1–3.7]
Crawford type				
Type I	2 (9.5)	1 (8.3)	1 (11.1)	1.375 [0.74–25.43]
Type II	7 (33.3)	3 (25)	4 (44.4)	2.4 [0.38–15.32]
Type III	7 (33.3)	5 (41.7)	2 (22.2)	0.4 [0.57–2.8]
Type IV	5 (23.8)	3 (25)	2 (22.2)	0.86 [0.11–6.62]
Max. aortic diameter (cm)	5.8 ± 1.5	6.3 ± 1.3	5.1 ± 1.5	−0.4 (*p* = 0.67) ^†^
Duration of surgery (min)	520 ± 89.6	490.5 ± 89.7	550 ± 85.1	0.3 (*p* = 0.19) ^†^
Cross-clamping time (min)	150.6 ± 36.4	147.8 ± 41.8	155.1 ± 29.5	0.1 (*p* = 0.66)

**Table 2:** Postoperative data. Values are the number (%) or mean ± SD, as indicated. Correlations are reported with Pearson’s (r). Significance is marked with (*) for *p* < 0.05 and (**) for *p* < 0.01.

	All (*n* = 21)	No Abdominal Complications (*n* = 12)	Abdominal Complications (*n* = 9)	Pearson’s r	*p*-Value
Thirty-day mortality	4 (19)	1 (8.3)	3 (33.3)	0.32	0.164
Hospital stay (days)	44.5 ± 32.7	30.9 ± 15.6	62.7 ± 41.1	0.49	0.02 *
Intensive Care Unit stay (days)	26.1 ± 25.4	17.5 ± 16	37.7 ± 31.6	0.4	0.07
Reintervention	11 (52.4)	4 (33.3)	7 (77.8)	0.44	0.046 *
- Re-thoracotomy	6 (28.6)	3 (25)	3 (33.3)	0.16	0.502
- Re-laparotomy	4 (19)	0	4 (44.4)	0.62	0.003 **
- Intestinal resection	2 (9.5)	0	2 (22.2)	−0.11	0.622
- Wound complications	3 (14.3)	2 (16.6)	1 (11.1)	−0.04	0.863
Time to abdominal complications (days)	4.8 ± 1.3				
Abdominal complications (total)	9 (42.9)	0 (0)	9 (100)		
- Paralytic ileus	2 (9.5)	0 (0)	2 (22.2)		
- Abdominal compartment syndrome	2 (9.5)	0 (0)	2 (22.2)		
- Visceral malperfusion	5 (23.8)	0 (0)	5 (55.6)		
Postop days to abd. complication (days)	1.9 ± 3.3	0 (0)	4.4 ± 3.9		
Sepsis	7 (33.3)	2 (16.7)	5 (55.6)	0.41	0.066
Multi-organ dysfunction syndrome	4 (19)	1 (8.3)	3 (33.3)	0.32	0.164
Acute kidney injury	13 (61.9)	6 (50)	7 (77.8)	0.28	0.214
Liver failure	4 (19)	1 (8.3)	3 (33.3)	0.32	0.164
Acute respiratory distress syndrome	9 (42.9)	4 (33.3)	5 (55.6)	0.22	0.333
Need for catecholamines (days)	11 ± 10.8	6 ± 6.4	17.9 ± 12	0.56	0.013 *

**Table 3:** Heat-shock protein 70 serum levels (ng/mL) reported as median [interquartile range]. ^†^ Adjusted *p*-values after Friedman analysis with Bonferroni correction for comparison to baseline levels. ^††^ *p*-values for correlations with abdominal complications (no vs. yes) are calculated with Dunn’s post hoc test for multiple comparisons after the Kruskal–Wallis nonparametric test. Significance marked with (*) for *p* < 0.05, (**) for *p* < 0.01, and (***) for *p* < 0.001.

	All (*n* = 21)	Adjusted *p*-Value ^†^	No Abdominal Complications (*n* = 12)	Adjusted *p*-Value ^†^	Abdominal Complications (*n* = 9)	Adjusted *p*-Value ^†^	*p*-Value ^††^
Baseline	0 [0–0]		0 [0–0]		0 [0–0]		0.92
Directly postoperative	11.77 [6.1–20.8]	<0.001 ***	9.5 [3.9–13.3]	0.002 **	18.3 [9.5–38]	0.014 *	0.1
12 h	7.59 [0–14]	0.002 **	0 [0–6.5]	0.796	14.5 [8.8–75.3]	0.003 **	0.007 **
24 h	1.33 [0–5.4]	0.357	0 [0–1.8]	1	6 [0.3–58.9]	0.526	0.15
48 h	0 [0–1.2]	1	0 [0–0]	1	1.2 [0–28]	1	0.06

**Table 4:** Spearman’s correlation for heat-shock protein 70 serum levels and the abdominal complications subgroups. Correlation presented with Spearman’s (rho). Significance marked with (*) for *p* < 0.05.

	Visceral Malperfusion (*n* = 5)	*p*-Value	Abdominal Compartment Syndrome (*n* = 2)	*p*-Value	Paralytic Ileus (*n* = 2)	*p*-Value
baseline	0.22	0.35	−0.11	0.65	−0.11	0.65
directly postoperatively	0.3	0.19	0.35	0.12	−0.13	0.56
12 h	0.43	0.05 *	0.41	0.07	−0.19	0.41
24 h	0.3	0.22	0.08	0.75	−0.36	0.12
48 h	0.34	0.14	0.14	0.57	−0.41	0.08

**Table 5:** ROC analysis for heat-shock protein 70 (HSP70) and lactate serum levels at different postoperative time points for patients with abdominal complications (*n* = 9). Cut-off was calculated with the Kolmogorov–Smirnov test. Significance marked with (*) for *p* < 0.05 and (**) for *p* < 0.01.

		AUC	*p*-Value	Cut-Off	Sensitivity (%)	Specificity (%)
HSP70 (ng/mL)	baseline	0.556	0.68	1.2	11.1	100
	directly postoperatively	0.788	0.03 *	14.4	66.7	83.3
	12 h	0.909	0.002 **	7.6	88.9	83.3
	24 h	0.788	0.03 *	3.3	66.7	100
	48 h	0.808	0.02 *	0.4	66.7	90.9
Lactate (mmol/L)	baseline	0.540	0.765	0.45	88.9	33.3
	directly postoperatively	0.667	0.188	2.65	66.7	66.7
	12 h	0.621	0.347	2.05	77.8	50
	24 h	0.687	0.141	1.55	77.8	78.7
	48 h	0.616	0.370	1.7	33.3	90.9

## Data Availability

The datasets generated and analyzed during the current study are available from the corresponding author on reasonable request.

## Supplementary Materials

The following supporting information can be downloaded at: https://www.mdpi.com/article/10.3390/ijms232315063/s1.
