# Supplementary material for: Onset of Adverse Abdominal Events Due to Intestinal Ischemia-Reperfusion Injury after Aortic Cross-Clamping Is Associated with Elevated HSP70 Serum Levels in the Early Postoperative Phase
Source: Int J Mol Sci. 2022 Dec 1;23(23):15063. doi: 10.3390/ijms232315063 (PMC9736142; doi:10.3390/ijms232315063)

Supplementary Table S1: Pearson's correlation with Firth's bias correction for heat-shock protein 70 serum levels and aortic cross-clamping time.

	Pearson's r	*p*-Value
baseline	−0.05	0.82
directly postoperatively	0.26	0.26
12 h	0.36	0.11
24 h	0.3	0.2
48 h	0.27	0.25

Supplementary Figure S1: Spaghetti plot of the course of heat-shock protein 70 (HSP70) serum levels during the first 48 hours after surgery.

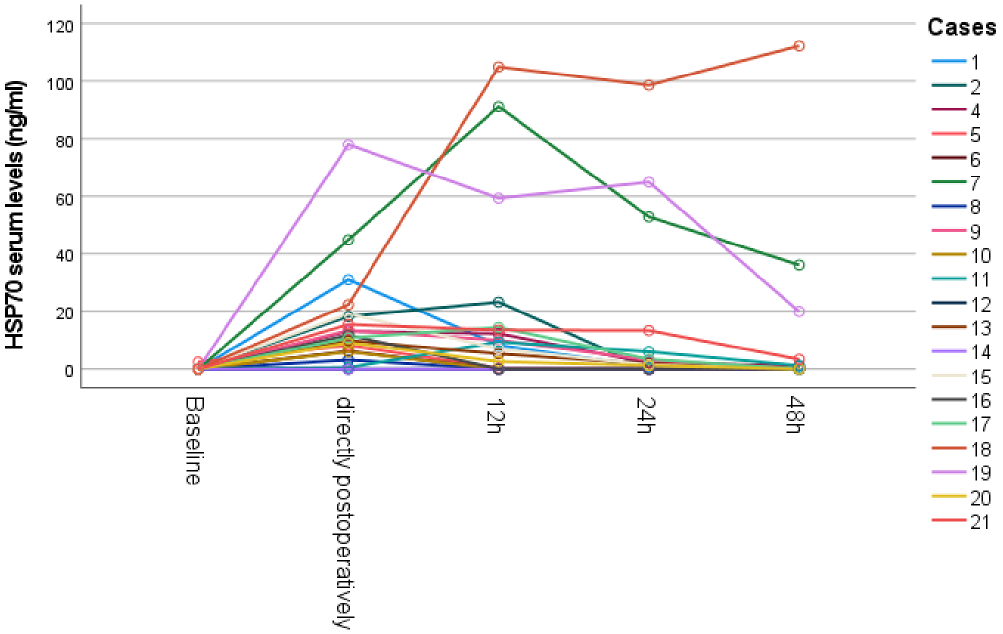

Supplementary Figure S2: Box-plot of heat-shock protein 70 (HSP70) serum levels 12 hours after surgery in relation to visceral malperfusion. Box represents the interquartile range with median and the whiskers represent minimum and maximum values. Mean is indicated with a cross and significance is annotated with (*) for *p*<.05.

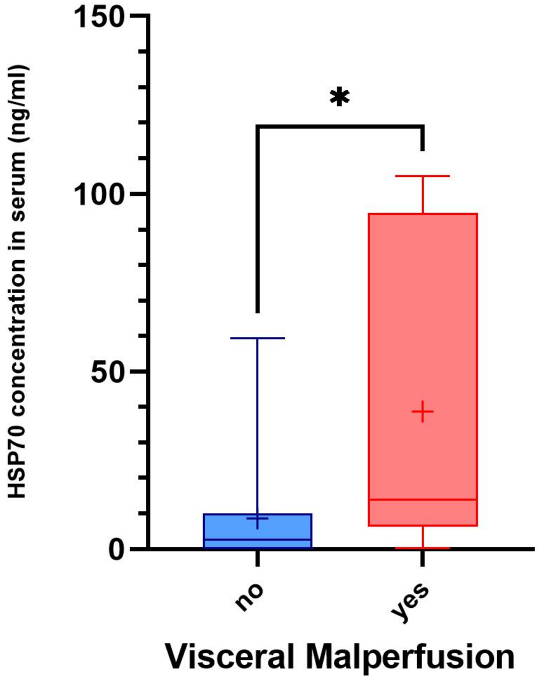

Supplement: Supplementary file 1 [file ijms-23-15063-s001.zip › ijms-1982953-supplementary.pdf]
